# Reliability of a musculoskeletal profiling test battery in elite academy soccer players

**DOI:** 10.1371/journal.pone.0236341

**Published:** 2020-07-23

**Authors:** Neval Grazette, Scot McAllister, Chin Wei Ong, Caroline Sunderland, Mary E. Nevill, John G. Morris

**Affiliations:** 1 Department of Sport Science, Sport, Health and Performance Enhancement Research Centre, School of Science and Technology, Nottingham Trent University, Nottingham, Nottinghamshire, United Kingdom; 2 Performance Services and Applied Research, Global Football, City Football Group, Manchester, United Kingdom; University of Belgrade, SERBIA

## Abstract

The study aimed to quantify the measurement error / reliability of a musculoskeletal profiling test battery administered in young, elite academy soccer players, and to examine if the order in which the test battery was administered, and who it was administered by, influenced reliability. Players (n = 75; age 12–20 years; stature 1.47–1.95 m; body mass 36–89 kg) from U-12 to U-23 age groups were assigned to either: 1) intra-rater-fixed order; 2) intra-rater-non-fixed order; 3) inter-rater-fixed order; or, 4) inter-rater-non-fixed order groups. On two separate occasions separated by 3 to 7 days, 12 raters conducted a musculoskeletal profiling test battery comprising 10 tests (Supine Medial Hip Rotation, Supine Lateral Hip Rotation, Hamstring 90/90, Prone Medial Hip Rotation [degrees]; Combined Elevation, Thoracic Rotation, Weight-Bearing Dorsiflexion, Y-Balance [centimetres]; Beighton, Lumbar Quadrant [categorical]). The measurement error / reliability for tests measured in degrees and centimetres was evaluated using the intraclass correlation (relative reliability), coefficient of variation and ratio limits of agreement (absolute reliability). Intraclass correlations varied from 0.04 (“poor”) to 0.95 (“excellent”), coefficient of variation from 2.9 to 43.4%, and the ratio limits of agreement from 1.058 (*/÷ 1.020) to 2.026 (*/÷ 1.319) for the tests measured in degrees and centimetres. The intraclass correlation, coefficient of variation and ratio limits of agreement were smallest for five out of eight tests measured in degrees and centimetres when the tests were administered in an intra-rater-fixed test order. These findings emphasise that different testing methods, and the administration of a musculoskeletal profiling test battery using a less than optimal design, will influence measurement error and hence test reliability. These observations need to be considered when investigating musculoskeletal function and age, injury, training or asymmetry in young, elite academy soccer players.

## Introduction

Young, elite academy soccer players undergo substantial anthropometric and physiological changes as they grow and mature [1]. Their involvement in training and competition in a high-intensity contact sport such as soccer is also physically demanding and presents a risk for injury [2]. Against this background of interacting physical change and sporting challenge, musculoskeletal profiling tests are often conducted on young players by academy medical and sports science staff at soccer clubs to monitor functional change with age or across a season, and to detect the functional characteristics or risk factors that predispose players to injury [3–6]. Musculoskeletal profiling tests may also be used for: monitoring the impact of injury and the progress of recovery; for examining asymmetrical differences in musculoskeletal function; and for assessing the effect of training [3, 7]. Additionally, the commitment to the musculoskeletal profiling of young players by academies and their staff ensures that professional, ethical and medico-legal obligations toward these non-adults are met [8]. Given the time, effort and resources often expended in many soccer academies on assessing the musculoskeletal profile of their players, it is important that the reliability of the musculoskeletal testing procedures they utilize is both carefully quantified and also deemed to be adequate for the purposes to which the tests are intended.

It is vital that measurements resulting from a musculoskeletal testing procedure are adequately reliable. Otherwise, a difference between two measurements could be assumed to indicate genuine change when in fact it is the result of too much measurement error or effectively inadequate reliability. Reliability is defined as the consistency of measurements, but it can also be considered as the amount of measurement error deemed acceptable for practical use, as some measurement error is always present when collecting data [9]. Importantly, this means that the assessment of reliability is not based on achieving a specific absolute boundary or value when applying some statistical procedure to some appropriate data; the assessment of reliability is actually context specific. So, given an amount of measurement error established using an appropriate statistical procedure (e.g. intra-class correlation, coefficient of variation based on differences, systematic bias ratio and the random error components of the 95% ratio limits of agreement), the reliability of a particular musculoskeletal test in one context (for example, measuring annual differences in a particular musculoskeletal function in a young player) may be deemed inadequate, whereas, in another context (for example, assessing the same particular musculoskeletal function before and following an injury), the identical test measurement error may be more than precise enough for the purpose and hence be deemed reliable.

Previously reported intra- and inter-rater reliability studies of musculoskeletal profiling test batteries in young individuals have primarily investigated general, symptomatic, non-soccer or sub-elite groups, suggesting there is a paucity of research evidencing the reliability of musculoskeletal profiling test batteries among young elite soccer players [10–14]. Of the two studies that have evaluated the reliability of musculoskeletal profiling test batteries in elite youth soccer players, the intraclass correlations for the tests in the batteries ranged from 0.51 (“fair”) to 0.98 (“excellent”), while the coefficient of variations ranged from 0.4 to 12.4% [4, 15]. However, Fourchet and colleagues only used 10 participants, of whom only four were soccer players [4], while Sporis and colleagues only used older soccer players [mean age of 18.1 years] [15]. Given the variety of movements required in a sport such as soccer and the fact that musculoskeletal function is joint specific, only a ‘battery’ of tests is likely to produce a sufficiently complete profile of the musculoskeletal function of players [16]. Therefore, the ubiquity of musculoskeletal profile testing in young, elite soccer players in applied settings such as academies, and the lack of studies investigating the reliability of a comprehensive musculoskeletal profile test battery in this specific sporting population and environment, suggests there is a gap in the research literature that needs to be addressed.

In order to minimise potential sources of measurement error, testing batteries are best conducted in a set order. For example, active and passive joint mobilisation can improve subsequent joint function (due to connective tissue changes that alter muscle length and joint kinematics), so testing Supine Medial Hip Rotation directly before testing Prone Medial Hip Rotation, is likely to elicit improved function in the second test compared to what might have been measured if the Prone Medial Hip Rotation was assessed first and / or in isolation [17, 18]. Similarly, potential measurement error is likely to be attenuated if the staff conducting such musculoskeletal tests do not vary. The practical challenges presented by an elite sporting environment such as a soccer academy, where hundreds of young players and many staff will have demanding training and match schedules, may mean that the staff conducting a musculoskeletal profiling testing battery, and the order in which the players complete the tests comprising the battery, may vary between testing occasions. Obviously, one wants to adopt the optimal testing design, which theoretically would be using the same staff to conduct all musculoskeletal profiling tests in a fixed order (“intra-rater-fixed order”), as this minimises the increase in measurement error likely to arise from using different staff and a test order which is not ‘fixed’. However, in a practical environment there may have to be a balance between what is theoretically optimal, versus what may be practically possible [19]. If it is deemed necessary to deviate from the ideal test order (such as adopting an “intra-rater-non-fixed”, “inter-rater-fixed”, or “inter-rater-non-fixed” testing order) for practical or pragmatic reasons, what is important is understanding the implications of adopting alternative testing designs, and quantifying the magnitude of any differences that may exist between these and the ideal, particularly where such alternative testing approaches may be what is actually most convenient and perhaps most likely to be used in many practical situations. Therefore, there does seem to be a need to examine how variation in the test order and variation in who is administering the tests may influence the reliability of a musculoskeletal profiling test battery in young, elite academy soccer players, as currently this is unknown.

Therefore, the aims of the current study were to: (i) quantify the relative and absolute measurement error / reliability of a battery of musculoskeletal profiling tests when administered in a sample of young, elite soccer players; and (ii) examine if the order in which the battery of tests was administered (fixed or non-fixed), and who it was administered by (intra- or inter-rater), influenced measurement error / reliability, and hence to help quantify the consequences of deviating from a theoretically ‘ideal’ intra-rater-fixed test order when conducting a musculoskeletal profiling test battery on young, elite soccer players.

## Methods

### Participants

Seventy-five elite youth soccer players from the ‘Youth Development’ (n = 52; U-12, U-13, U-14, U-16 age groups; mean ± SD [range]: age 14.0 ± 1.1 [11.8–15.7] years; stature 1.66 ± 0.12 [1.47–1.95] metres; body mass 54.5 ± 12.1 [36.0–78.0] kilograms) and ‘Professional Development’ (n = 23; U-18 and U-23 age groups; age 18.0 ± 0.9 [16.3–19.8] years; stature 1.78 ± 0.06 [1.66–1.91] metres; body mass 72.8 ± 8.1 [58.4–89.0] kilograms) phases participated in the present study during the 2017–2018 season. All participants were registered at a full-time category one soccer academy in England, which is operated by a professional soccer club. The category one academy is governed by the Elite Player Performance Plan (EPPP) of the English Premier League, which represents the highest possible level of youth soccer in England, therefore, players within the present study were deemed to be ‘elite’ [20, 21]. All participants engaged in 3–4 training days (1–3 sessions per day) and 1–2 competitive matches per week. Following consent from the academy, ethical approval was obtained from the University Ethics Committee. Participants were familiar with the musculoskeletal profiling test battery within the reliability study as it was conducted as part of their regular monitoring. Players or parents / guardians provided written informed consent and assent, depending on the age of the participant. All data utilised in the study was anonymised by the academy prior to analysis.

### Raters

Twelve experienced raters were tested for intra- and inter-rater reliability. Six of the raters were part of the sports medicine team (5 chartered physiotherapists and 1 performance therapist), and they averaged 16 years of clinical experience. The other six raters were part of the sports science team (6 sport scientists / strength and conditioning coaches) and they averaged 8 years of applied experience. All raters were trained on how to conduct each musculoskeletal profiling test by an experienced chartered physiotherapist. The physiotherapist demonstrated the appropriate measurement techniques, and then the rater practiced on an individual until the experienced physiotherapist deemed their measurement technique appropriate. Additionally, detailed written and video procedure manuals developed by the sports medicine team for the test battery were provided prior to testing, and were always available for consultation.

### Procedure

The musculoskeletal profiling test battery was compiled by the sports medicine team based on their clinical judgement, to assess joint ROM of the upper and lower limbs. Participants were assigned to four groups (A, B, C, D) that were randomly allocated to either: 1) intra-rater-fixed order; 2) intra-rater-non-fixed order: 3) inter-rater-fixed order; or, 4) inter-rater-non-fixed order groups. Each participant group comprised a variety of players from the U-12 to U-23 age groups (see S1 and S2 Tables). Fixed order groups completed the musculoskeletal profiling test battery in a predetermined sequential order (1. Supine Medial Hip Rotation; 2. Supine Lateral Hip Rotation; 3. Hamstring 90/90; 4. Prone Medial Hip Rotation; 5. Beighton; 6. Lumbar Quadrant; 7. Combined Elevation; 8. Thoracic Rotation; 9. Weight-Bearing Dorsiflexion; 10. Y-Balance), based on the practical reasoning of the sports medicine team, while the non-fixed test order groups completed the test battery as they typically would in a practical situation, based on testing station availability. Groups A to D were unchanged for the initial six tests, but participant groups switched experimental conditions for the remaining four tests (Tables 1–3) due to unexpected practical challenges (see limitations paragraph at the end of discussion). An observation of the non-fixed testing order strategies was conducted on 31 participants during a subsequent routine testing occasion, and reported 23 different non-fixed test orders combinations, with none of the participants completing the fixed test order used in the present study. Musculoskeletal profiling tests within the 10-test battery were grouped according to their respective measurement units (Degrees [4-tests], Centimetres [4-tests] and Categorical [2-tests]) to coherently report findings. Categorical measurements and tests measured in degrees were conducted by the sports medicine team only, as these tests were considered more suited to be performed by clinical practitioners as they require manual handling skills and appropriate training, while both the sports medicine and sports science / strength and conditioning teams conducted tests recorded in centimetres. On two separate occasions separated by 3 to 7 days, the 12 raters conducted the musculoskeletal profiling test battery under the same environmental conditions, and at the same exact times to control for diurnal variations. Verbal instructions and demonstrations were conducted by raters prior to, and during testing. All tests were conducted in a gym and the sports medicine treatment areas at different testing stations, and participants wore club issued training clothing. Raters did not have access to the results from the previous testing occasion (test 1), as the subsequent testing occasion (test 2) results were recorded on a separate sheet. Bilateral measurements were recorded for all tests and treated as separate measures (1 participant: n = 2), except for the Beighton (lumbar flexion) and Combined Elevation (1 participant: n = 1) tests.

**Table 1:** Absolute and relative reliability measures for musculoskeletal profiling tests measured in degrees.

	Intra-Rater Reliability		Inter-Rater Reliability	
	**Supine Medial Hip Rotation**			
	**Fixed (n = 52) [A]**	**Non-Fixed (n = 48) [B]**	**Fixed (n = 28) [C]**	**Non-Fixed (n = 22) [D]**
test 1 (X±SD) [°]	35.0 ± 9.2	35.6 ± 8.9	37.0 ± 14.0^1^	32.0 ± 11.4
test 2 (X±SD) [°]	36.8 ± 12.0	37.2 ± 9.6	45.1 ± 7.3	40.2 ± 7.7
test 1 (X±SD) [ln]	3.518 ± 0.286	3.537 ± 0.290	3.545 ± 0.371	3.400 ± 0.372
test 2 (X±SD) [ln]	3.556 ± 0.322	3.583 ± 0.264	3.796 ± 0.167	3.676 ± 0.203
systematic bias (*/÷CI)	1.039 (*/÷ 1.066)	1.047 (*/÷ 1.065)	1.285 (*/÷ 1.135)	1.317 (*/÷ 1.173)
ratio LoA (*/÷CI)	1.563 (*/÷ 1.117)	1.527 (*/÷ 1.115)	1.894 (*/÷ 1.245)	2.026 (*/÷ 1.319)
ICC (CI)	0.72 (0.56–0.83)	0.69 (0.51–0.81)	0.26 (-0.06–0.56)	0.20 (-0.12–0.53)
t-test	P = 0.236	P = 0.148	P<0.001^×^	P = 0.002^×^
cohen’s d	0.13 (trivial)	0.17 (trivial)	0.87 (large)	0.92 (large)
variationLoA [°]	35.9: 23.9, 58.3	36.4: 25.0, 58.2	41.1: 27.9, 100.0	36.1: 23.5, 96.3
variationCV [°]	35.9: 26.7, 45.1	36.4: 27.7, 45.1	41.1: 25.3, 56.9	36.1: 20.4, 51.8
	**Supine Lateral Hip Rotation**			
	**Fixed (n = 52) [A]**	**Non-Fixed (n = 48) [B]**	**Fixed (n = 28) [C]**	**Non-Fixed (n = 22) [D]**
test 1 (X±SD) [°]	49.4 ± 10.6	49.6 ± 11.4	48.5 ± 10.7	51.6 ± 11.4
test 2 (X±SD) [°]	48.5 ± 12.0	49.3 ± 12.7	57.2 ± 9.8	63.5 ± 8.7
test 1 (X±SD) [ln]	3.878 ± 0.216	3.879 ± 0.230	3.857 ± 0.232	3.920 ± 0.226
test 2 (X±SD) [ln]	3.852 ± 0.250	3.868 ± 0.247	4.032 ± 0.175	4.142 ± 0.142
systematic bias (*/÷CI)	0.974 (*/÷ 1.040)	0.988 (*/÷ 1.047)	1.191 (*/÷ 1.104)	1.238 (*/÷ 1.072)
ratio LoA (*/÷CI)	1.312 (*/÷ 1.070)	1.360 (*/÷ 1.082)	1.650 (*/÷ 1.187)	1.361 (*/÷ 1.128)
ICC (CI)	0.82 (0.71–0.89)	0.79 (0.65–0.87)	0.17 (-0.12–0.47)	0.43 (-0.11–0.77)
t-test	P = 0.181	P = 0.612	P = 0.001^×^	P<0.001^×^
cohen’s d	0.11 (trivial)	0.05 (trivial)	0.85 (large)	1.18 (large)
variationLoA [°]	49.0: 36.4, 62.6	49.5: 36.0, 66.5	52.8: 38.1, 103.9	57.6: 52.4, 97.0
variationCV [°]	49.0: 41.6, 56.4	49.5: 41.2, 57.8	52.8: 37.3, 68.3	57.6: 48.8, 66.4
	**Hamstring 90/90**			
	**Fixed (n = 36) [A]**	**Non-Fixed (n = 38) [B]**	**Fixed (n = 28) [C]**	**Non-Fixed (n = 22) [D]**
test 1 (X±SD) [°]	60.4 ± 12.4	66.1 ± 13.5	67.3 ± 9.0	70.2 ± 13.5
test 2 (X±SD) [°]	70.3 ± 10	72.8 ± 10.8	79.3 ± 8.7	77.0 ± 10.9
test 1 (X±SD) [ln]	4.081 ± 0.207	4.166 ± 0.242	4.200 ± 0.134	4.233 ± 0.193
test 2 (X±SD) [ln]	4.242 ± 0.143	4.275 ± 0.167	4.367 ± 0.116	4.334 ± 0.141
systematic bias (*/÷CI)	1.175 (*/÷ 1.051)	1.115 (*/÷ 1.061)	1.182 (*/÷ 1.068)	1.105 (*/÷ 1.064)
ratio LoA (*/÷CI)	1.334 (*/÷ 1.090)	1.416 (*/÷ 1.107)	1.397 (*/÷ 1.122)	1.316 (*/÷ 1.114)
ICC (CI)	0.47 (-0.06–0.76)	0.56 (0.22–0.77)	0.04 (-0.14–0.28)	0.57 (0.12–0.81)
t-test	P<0.001^×^	P = 0.001^×^	P<0.001^×^	P = 0.003^×^
cohen’s d	0.91 (large)	0.52 (medium)	0.98 (large)	0.72 (medium)
variationLoA [°]	65.3: 57.6, 102.4	69.4: 54.7, 109.6	73.3: 62.0, 121.0	69.8: 58.6, 101.6
variationCV [°]	65.3: 54.9, 75.7	69.4: 55.8, 83.0	73.3: 59.7, 86.9	69.8: 59.3, 80.3
	**Prone Medial Hip Rotation**			
	**Fixed (n = 52) [A]**	**Non-Fixed (n = 48) [B]**	**Fixed (n = 28) [C]**	**Non-Fixed (n = 22) [D]**
test 1 (X±SD) [°]	36.6 ± 9.0	35.8 ± 10.6	24.9 ± 6.1^1^	25.6 ± 6.3
test 2 (X±SD) [°]	36.4 ± 7.2	35.9 ± 7.9	30.0 ± 8.0	27.3.± 7.6
test 1 (X±SD) [ln]	3.563 ± 0.295	3.520 ± 0.372	3.182 ± 0.256^1^	3.213 ± 0.251
test 2 (X±SD) [ln]	3.573 ± 0.208	3.558 ± 0.222	3.362 ± 0.296^1^	3.268 ± 0.291
systematic bias (*/÷CI)	1.011 (*/÷ 1.057)	1.038 (*/÷ 1.070)	1.197 (*/÷ 1.094)^1^	1.057 (*/÷ 1.108)^1^
ratio LoA (*/÷CI)	1.478 (*/÷ 1.102)	1.580 (*/÷ 1.125)	1.571 (*/÷ 1.167)	1.573 (*/÷ 1.194)
ICC (CI)	0.70 (0.53–0.82)	0.71 (0.53–0.83)	0.55 (0.10–0.79)	0.64 (0.31–0.83)
t-test	P = 0.701	P = 0.270	P = 0.038^×^	P = 0.481
cohen’s d	0.04 (trivial)	0.12 (trivial)	0.65 (medium)	0.20 (small)
variationLoA [°]	36.5: 24.9, 54.5	35.8: 23.6, 58.8	27.4: 20.9, 51.6	26.5: 17.8, 44.0
variationCV [°]	36.5: 28.4, 44.6	35.8: 26.4, 45.2	27.4: 20.2, 34.6	26.5: 19.6, 33.4

A, B, C, D = different participant groups; X±SD = mean ± standard deviation;

° = degrees;

ln = logarithm transformed; LoA = limits of agreement; ICC = intraclass correlation; CI = 95% confidence interval;

^1^ = p<0.05, not normally distributed (Shapiro-Wilk test of normality);

^×^ = p<0.05, significant difference between test 1 vs test 2;

variationLoA = example of possible variation of measurement based on the ratio LoA; variationCV = example of possible variation of measurement based on the coefficient of variation.

**Table 2:** Absolute and relative reliability measures for musculoskeletal profiling tests measured in centimetres.

	Intra-Rater Reliability		Inter-Rater Reliability	
	**Combined Elevation**			
	**Fixed (n = 14) [C]**	**Non-Fixed (n = 11) [D]**	**Fixed (n = 26) [A]**	**Non-Fixed (n = 23) [B]**
test 1 (X±SD) [cm]	14.8 ± 5.1	15.6 ± 5.6	15.5 ± 5.1	14.2 ± 3.9
test 2 (X±SD) [cm]	14.7 ± 5.6	16.2 ± 5.3	13.6 ± 5.1^1^	13.1 ± 4.5^1^
test 1 (X±SD) [ln]	2.630 ± 0.395	2.683 ± 0.382	2.688 ± 0.334	2.611 ± 0.288
test 2 (X±SD) [ln]	2.608 ± 0.441	2.735 ± 0.342	2.552 ± 0.341	2.519 ± 0.331
systematic bias (*/÷CI)	0.979 (*/÷ 1.119)^1^	1.054 (*/÷ 1.096)	0.873 (*/÷ 1.095)	0.911 (*/÷ 1.113)
ratio LoA (*/÷CI)	1.466 (*/÷ 1.216)	1.305 (*/÷ 1.171)	1.552 (*/÷ 1.170)	1.622 (*/÷ 1.203)
ICC (CI)	0.90 (0.71–0.97)	0.93 (0.76–0.98)	0.73 (0.39–0.88)	0.66 (0.36–0.84)
t-test	P = 1.000	P = 0.231	P = 0.005^×^	P = 0.085
cohen’s d	0.05 (trivial)	0.14 (trivial)	0.40 (small)	0.30 (small)
variationLoA [cm]	14.8: 9.8, 21.2	15.9: 12.8, 21.9	14.5: 8.2, 19.7	13.6: 7.7, 20.1
variationCV [cm]	14.8: 11.6, 18.0	15.9: 13.6, 18.2	14.5: 10.9, 18.1	13.6: 9.8, 17.4
	**Thoracic Rotation**			
	**Fixed (n = 28) [C]**	**Non-Fixed (n = 22) [D]**	**Fixed (n = 52) [A]**	**Non-Fixed (n = 44) [B]**
test 1 (X±SD) [cm]	16.8 ± 4.6	18.8 ± 3.1	16.8 ± 4.5	14.3 ± 4.0
test 2 (X±SD) [cm]	16.6 ± 3.7	16.8 ± 4.4	16.8 ± 4.3	15.5 ± 5.0
test 1 (X±SD) [ln]	2.783 ± 0.302^1^	2.923 ± 0.169	2.788 ± 0.273	2.625 ± 0.267
test 2 (X±SD) [ln]	2.783 ± 0.226	2.777 ± 0.317^1^	2.788 ± 0.280	2.691 ± 0.329
systematic bias (*/÷CI)	1.001 (*/÷ 1.082)	0.864 (*/÷ 1.121)	1.000 (*/÷ 1.072)	1.068 (*/÷ 1.086)
ratio LoA (*/÷CI)	1.489 (*/÷ 1.146)	1.658 (*/÷ 1.219)	1.625 (*/÷ 1.128)	1.699 (*/÷ 1.153)
ICC (CI)	0.72 (0.47–0.86)	0.42 (0.04–0.71)	0.60 (0.40–0.75)	0.58 (0.35–0.75)
t-test	P = 0.987	P = 0.015^×^	P = 0.994	P = 0.112
cohen’s d	0.00 (trivial)	0.58 (medium)	0.00 (trivial)	0.22 (small)
variationLoA [cm]	16.7: 11.2, 24.9	17.8: 9.3, 25.5	16.8: 10.4, 27.4	14.9: 9.4, 27.1
variationCV [cm]	16.7: 12.9, 20.5	17.8: 12.6, 23.0	16.8: 12.0, 21.6	14.9: 10.2, 19.6
	**Weight-Bearing Dorsiflexion**			
	**Fixed (n = 28) [C]**	**Non-Fixed (n = 22) [D]**	**Fixed (n = 52) [A]**	**Non-Fixed (n = 46) [B]**
test 1 (X±SD) [cm]	8.6 ± 2.2^1^	8.3 ± 2.7	8.1 ± 2.2	7.0 ± 3.1
test 2 (X±SD) [cm]	8.1 ± 2.5^1^	7.1 ± 2.4	8.4 ± 2.4	7.2 ± 3.0
test 1 (X±SD) [ln]	2.085 ± 0.454^1^	2.038 ± 0.443^1^	2.048 ± 0.312	1.811 ± 0.591
test 2 (X±SD) [ln]	2.013 ± 0.474^1^	1.891 ± 0.407^1^	2.073 ± 0.348	1.878 ± 0.480
systematic bias (*/÷CI)	0.931 (*/÷ 1.055)^1^	0.864 (*/÷ 1.076)	1.025 (*/÷ 1.047)	1.068 (*/÷ 1.100)
ratio LoA (*/÷CI)	1.309 (*/÷ 1.097)	1.384 (*/÷ 1.136)	1.380 (*/÷ 1.083)	1.868 (*/÷ 1.179)
ICC (CI)	0.95 (0.86–0.98)	0.88 (0.46–0.96)	0.88 (0.79–0.93)	0.82 (0.70–0.90)
t-test	P = 0.049^×^	P<0.001^×^	P = 0.281	P = 0.165
cohen’s d	0.16 (trivial)	0.35 (small)	0.08 (trivial)	0.12 (trivial)
variationLoA [cm]	8.3: 5.9, 10.1	7.7: 4.8, 9.2	8.2: 6.1, 11.6	7.1: 4.1, 14.2
variationCV [cm]	8.3: 7.1, 9.5	7.7: 6.3, 9.1	8.2: 6.8, 9.6	7.1: 4.4, 9.8
	**Y-Balance**			
	**Fixed (n = 28) [C]**	**Non-Fixed (n = 22) [D]**	**Fixed (n = 50) [A]**	**Non-Fixed (n = 46) [B]**
test 1 (X±SD) [cm]	264.0 ± 17.2	263.8 ± 14.2^1^	245.2 ± 18.2	246.2 ± 16.0
test 2 (X±SD) [cm]	261.4 ± 16.7	266.1 ± 13.2	243.9 ± 18.0	241.9 ± 21.9
test 1 (X±SD) [ln]	5.574 ± 0.066	5.574 ± 0.053^1^	5.500 ± 0.073	5.485 ± 0.072
test 2 (X±SD) [ln]	5.564 ± 0.064	5.583 ± 0.050	5.494 ± 0.073	5.447 ± 0.097
systematic bias (*/÷CI)	0.990 (*/÷ 1.011)	1.009 (*/÷ 1.018)	0.995 (*/÷ 1.019)	0.980 (*/÷ 1.023)
ratio LoA (*/÷CI)	1.058 (*/÷ 1.020)	1.080 (*/÷ 1.031)	1.096 (*/÷ 1.033)	1.107 (*/÷ 1.040)
ICC (CI)	0.89 (0.78–0.95)	0.71 (0.42–0.87)	0.80 (0.67–0.88)	0.77 (0.60–0.87)
t-test	P = 0.095	P = 0.298	P = 0.435	P = 0.013^×^
cohen’s d	0.15 (trivial)	0.18 (trivial)	0.08 (trivial)	0.45 (small)
variationLoA [cm]	262.7: 245.9, 275.4	265.0: 247.6, 288.7	244.6: 222.0, 266.6	244.0: 216.1, 264.9
variationCV [cm]	262.7: 259.0, 266.4	265.0: 252.9, 277.1	244.6: 233.4, 255.8	244.0: 232.8, 255.2

A, B, C, D = different participant groups; X±SD = mean ± standard deviation; cm = centimetres; ln = logarithm transformed; LoA = limits of agreement; ICC = intraclass correlation; CI = 95% confidence interval;

^1^ = p<0.05, not normally distributed (Shapiro-Wilk test of normality);

^×^ = p<0.05, significant difference between test 1 vs test 2;

variationLoA = example of possible variation of measurement based on the ratio LoA; variationCV = example of possible variation of measurement based on the coefficient of variation.

**Table 3:** Reliability measures for the categorical tests within the musculoskeletal profiling test battery.

	Intra-Rater Reliability		Inter-Rater Reliability	
	**Beighton**			
	**Fixed (n = 26) [A]**	**Non-Fixed (n = 23) [B]**	**Fixed (n = 14) [C]**	**Non-Fixed (n = 11) [D]**
weighted kappa	0.51	0.64	0.46	0.33
percentage agreement	69%	74%	79%	82%
	**Lumbar Quadrant**			
	**Fixed (n = 36) [A]**	**Non-Fixed (n = 38) [B]**	**Fixed (n = 28) [C]**	**Non-Fixed (n = 22) [D]**
weighted kappa	0.47	0.08	0.73	0.40
percentage agreement	94%	79%	89%	77%

A, B, C, D = different participant groups.

### Musculoskeletal profiling test battery

#### Supine Medial Hip Rotation [°]

**Fig 1:**
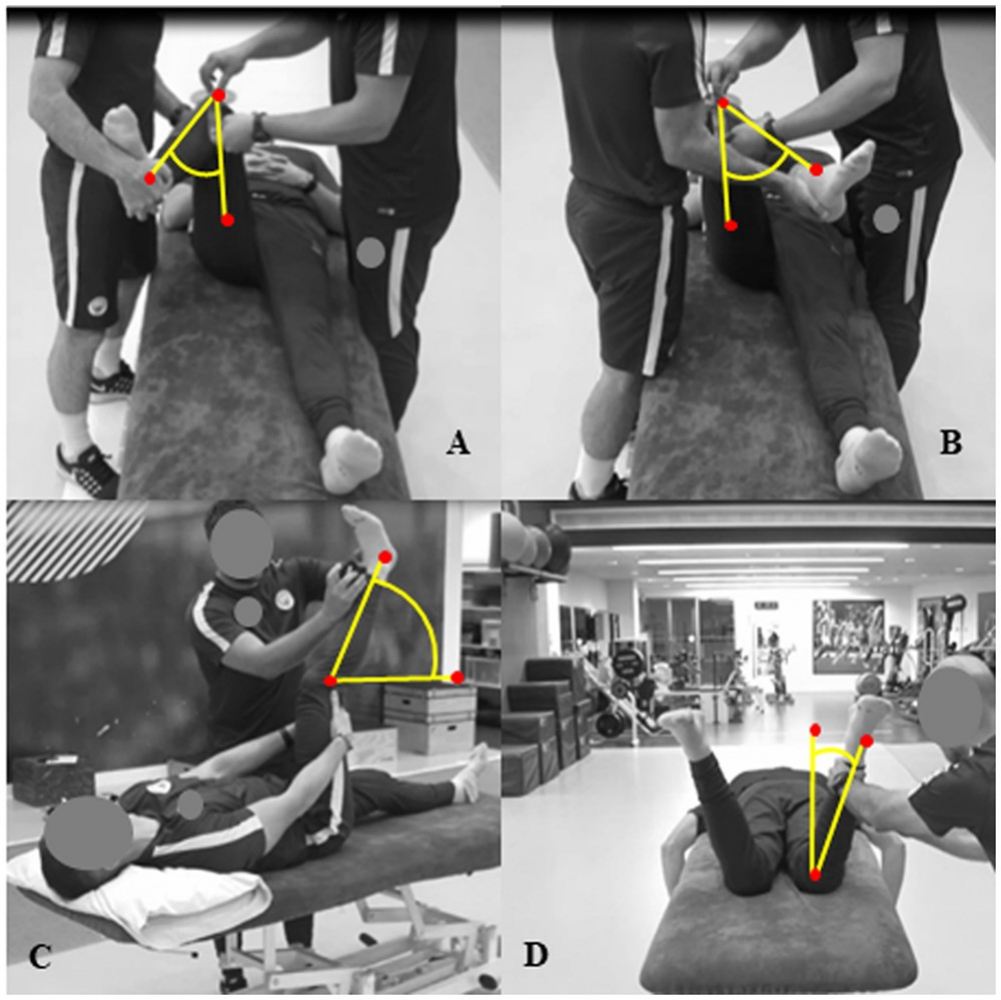
Visual representations of the musculoskeletal profiling tests measured in degrees. A: Supine Medial Hip Rotation; B: Supine Lateral Hip Rotation; C: Hamstring 90/90; D: Prone Medial Hip Rotation.

Participants were in a supine position, before a rater passively flexed the test leg hip and knee joints at 90°, while the contralateral leg was extended in a neutral position. Another rater placed the fulcrum of a 30.5 cm plastic manual goniometer (66fit, Physio Supplies Limited, Lincolnshire, UK) on the apex of the patella, with the movement arm placed on the midline of the tibia, while the stationary arm remained perpendicular to the floor. The rater then passively moved the lower leg to facilitate medial hip rotation to the point of resistance, and medial hip rotation was measured as the degrees of deviation from the starting position [22] (Fig 1A).

#### Supine Lateral Hip Rotation [°]

Participants were in a supine position, before a rater passively flexed the test leg hip and knee joints at 90°, while the contralateral leg was extended in a neutral position. Another rater placed the fulcrum of a 30.5 cm plastic manual goniometer (66fit, Physio Supplies Limited, Lincolnshire, UK) on the apex of the patella, with the movement arm placed on the midline of the tibia, while the stationary arm remained perpendicular to the floor. The rater then passively moved the lower leg to facilitate lateral hip rotation to the point of resistance, and lateral hip rotation was measured as the degrees of deviation from the starting position [22] (Fig 1B).

#### Hamstring 90/90 (active knee extension) [°]

Participants were in a supine position with the test leg’s knee and hip flexed at 90°, and both arms holding the posterior thigh to maintain the hip and knee 90° positions. Participants then actively fully extended the knee to the point of resistance while the contralateral leg remained extended in a neutral position, then a rater placed a digital goniometer (Acumar Digital Inclinometer; Lafayette Instrument Company, Indiana, USA) on the distal tibia to record the degrees of deviation from the starting position [23] (Fig 1C).

#### Prone Medial Hip Rotation [°]

While in a prone position with the hips in neutral, and both knees flexed at 90°, a rater guided both limbs to end range medial rotation, and each limb was measured individually as the degrees of deviation from the starting position with a digital goniometer (Acumar Digital Inclinometer; Lafayette Instrument Company, Indiana, USA) placed on the lateral border of the lower leg [12] (Fig 1D).

#### Combined elevation (thoracic extension and shoulder flexion) [cm]

**Fig 2:**
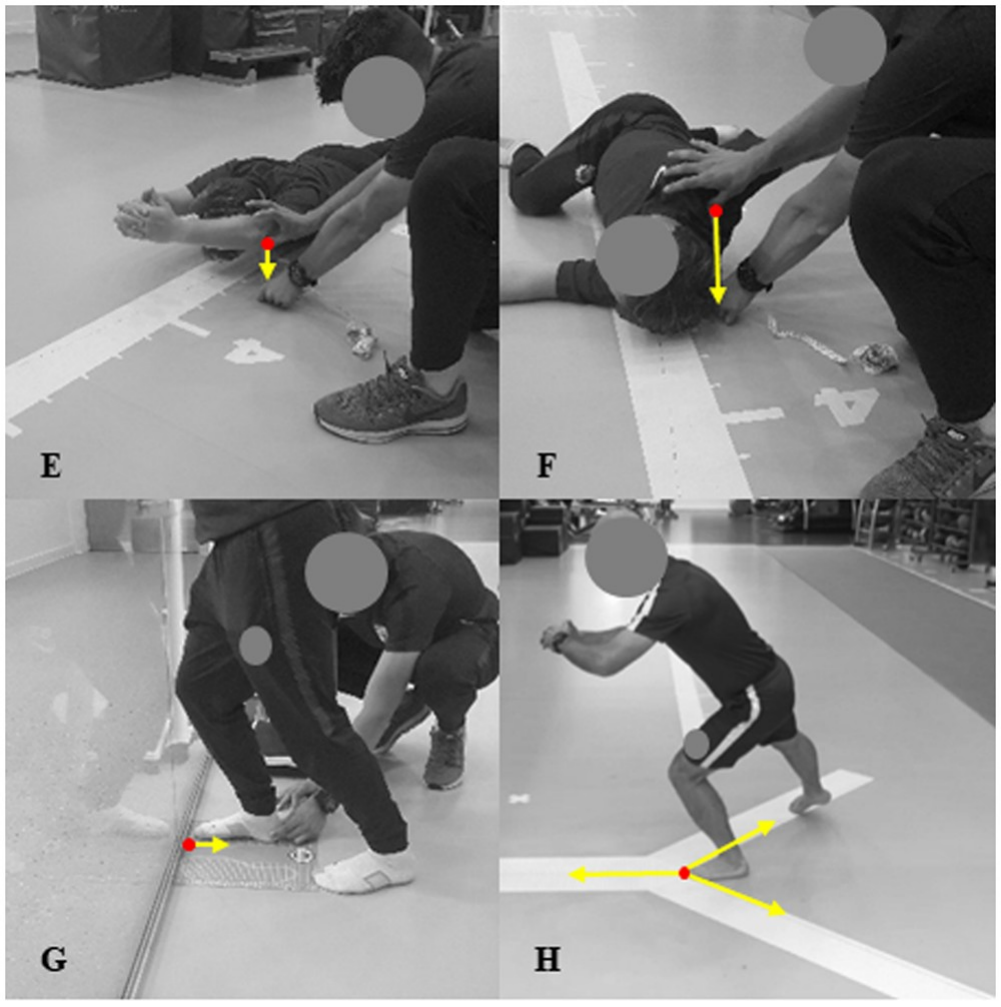
Visual representations of the musculoskeletal profiling tests measured in centimetres. E: Combined Elevation; F: Thoracic Rotation; G: Weight-Bearing Dorsiflexion; H: Y-Balance.

Participants assumed a prone position on the floor with arms outstretched above the head and feet together; elbows locked in full extension with interlaced fingers, and the chin resting on the floor. Participants were then instructed to lift both arms as far off the floor as possible with elbows extended; whilst keeping feet, knees, hips and chin in constant contact with the ground. At maximum ROM, the distance from the elbow to the floor was taken with a measuring tape from one side [24] (Fig 2E).

#### Thoracic Rotation [cm]

Participants were in a side lying position, shoulder aligned with the hips in neutral and both knees flexed at 90°. The upper leg then rotated across the hips, with the hip and knee bent at 90°, and the knee of the rotated leg in constant contact with floor. The arms were fully extended and stacked perpendicular to the trunk as both knees remained flexed at 90°, then the top arm rotated across the trunk as far as possible. At maximum ROM the distance from the acromioclavicular joint to the floor on the measurement side was recorded with a measuring tape (Fig 2F).

#### Weight-Bearing Dorsiflexion [cm]

Participants positioned their foot perpendicular to a wall so that it was aligned on the measurement surface on the floor. No shoes were worn, and participants were permitted to hold onto the wall for balance during the test, and were allowed to rest the untested contralateral leg in a comfortable position on the floor. During dorsiflexion, the participants’ heel was held by the rater to prevent it from lifting off the floor, while pronation or supination of the foot, along with pelvic rotation and knee valgus or varus were all verbally discouraged. Participants lunged their knee towards the wall until it touched, progressively moving their foot away from the wall. At maximum ankle dorsiflexion ROM while maintaining knee contact with the wall and heel contact with the floor, the distance recorded was taken from the big toe to the wall with the centimetres measurement units on the floor [25] (Fig 2G).

#### Y-Balance (dynamic balance and neuromuscular control) [cm]

Participants placed their big toe at the apex of the intersection of three lines that formed a “Y” shape on the floor. While descending into a unilateral squat, participants sequentially reached their contralateral leg in the anterior, posteromedial and posterolateral directions. Participants’ maximal reach was achieved with a light touch of the big toe, and the distance from the centre was recorded before returning to the start position. A measure was only recorded if the participant maintained constant heel contact and good postural control with the unilateral squat limb during each reach and return to neutral. A composite score of the three directions completed were used for analysis [26] (Fig 2H).

#### Beighton (joint hypermobility)

Raters sequentially took joint ROM measures for extension / hyperextension from the: little finger; thumb to wrist; elbow and knee; along with lumbar flexion. All tests were conducted with participants lying in a supine position, except for lumbar flexion, which was conducted in a standing position. Each test was scored based on the rater passively moving the joints into the desired positions, except for active lumbar flexion, without the use of any measurement devices. The subjective scores given were either a score of 1 (hypermobile) or 0 (not hypermobile) for each element of the test, and totalled for the combined score. Combined scores were then placed into 1 of 3 categories: not hypermobile (0 to 2); increased mobility (3 to 5); hypermobile (6 to 9) [27].

**Fig 3:**
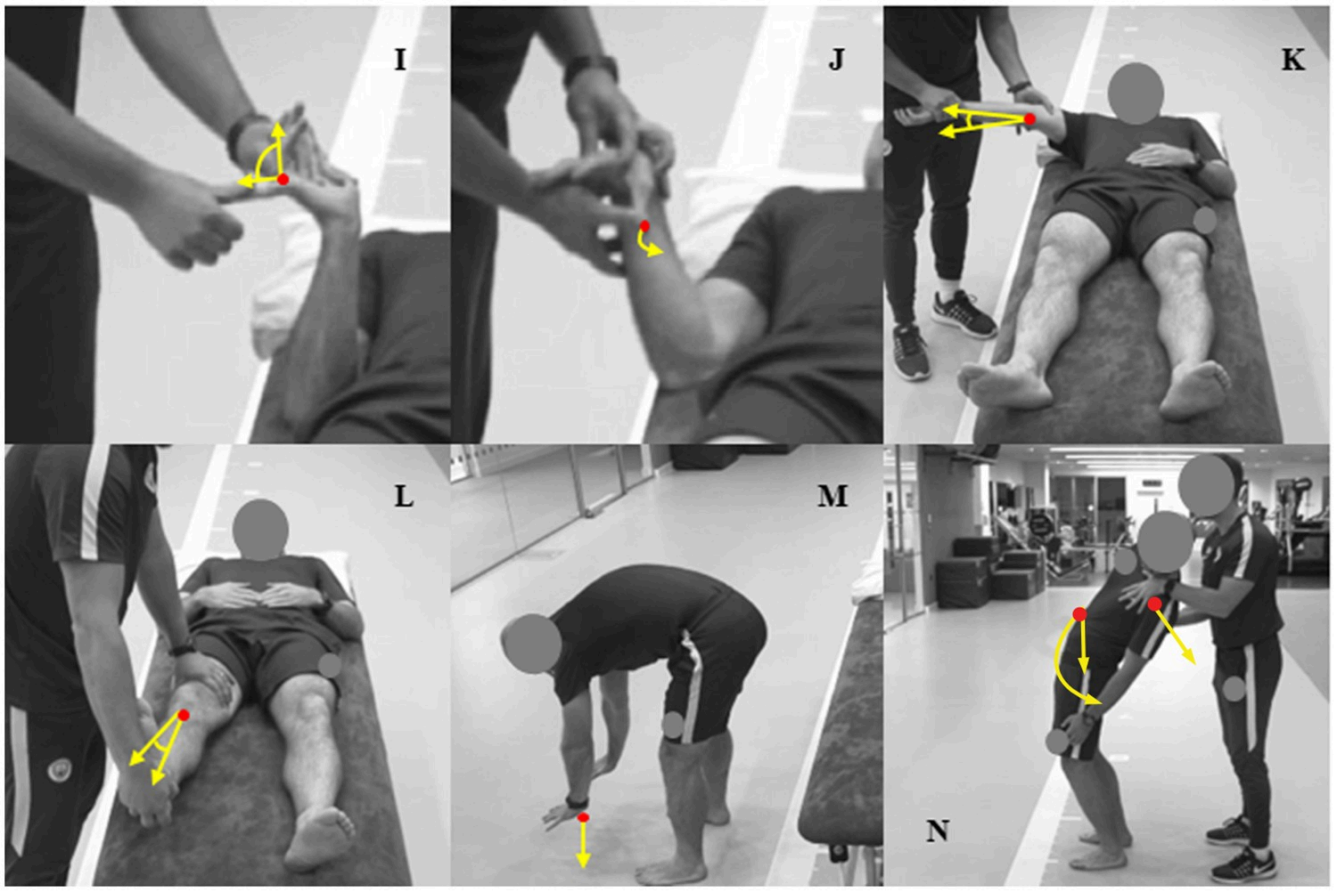
Visual representations of categorical measurements for the Beighton and Lumbar Quadrant musculoskeletal profiling tests. I: little finger; J: thumb to wrist; K: elbow extension; L: knee extension; M: lumbar flexion; N: Lumbar Quadrant.

**a)** little finger–The tip of the little finger was passively hyperextended pain free as far as possible by the rater using their thumb. Hyperextension (> 90°) resulted in a score of 1, while hyperextension of (≤ 90°) resulted in a score of 0 (Fig 3I).
**b)** thumb to wrist–With a flexed wrist, the tester passively abducted the thumb towards the radial aspect the forearm. If the thumb touched the forearm, a score of 1 was given, and if it did not touch, a score of 0 was given (Fig 3J).
**c)** elbow extension–The participant’s shoulder was abducted and the forearm supinated, with the proximal elbow stabilised from the posterior side by the rater. A gentle force was then applied to the participant’s palmar wrist to reach passive end range elbow extension. Hyperextension of the elbow (>10°) resulted in a score of 1, while hyperextension of the elbow (≤ 10°) resulted in a score of 0 (Fig 3K).
**d)** knee extension–Laid in a supine position, the superior aspect of the knee was anteriorly stabilised and gently extended by lifting the calcaneus. Knee hyperextension (>10°) resulted in a score of 1, while hyperextension of the knee (≤ 10°) resulted in a score of 0 (Fig 3L).
**e)** lumbar flexion–Participants attempted to touch the floor with their palms flat on the floor while maintaining knee extension or hyperextension. Lumbar flexion with palms completely flat on the ground, resulted in a score of 1; otherwise, a score of 0 was given (Fig 3M).

#### Lumbar Quadrant (restricted movement and / or local or referred pain)

While barefoot in a standing position with feet shoulder width apart, the rater placed one hand above the contralateral iliac crest to stabilise the hips, while the other hand was placed on the ipsilateral shoulder of the side being measured to guide the movement. Compressive forces were applied to the lumbar spine as participants were sequentially guided into a combination of end-range ipsilateral lumbar lateral flexion, extension and rotation, while maintaining balance on both feet. Raters used a 3-point numerical rating scale (1 = pain free and no stiffness; 2 = pain or stiffness; 3 = pain and stiffness) based on the participant’s response and the rater’s observational judgement [28] (Fig 3N).

### Statistical analyses

#### Tests measured in degrees and centimetres

Data were analysed using the Statistical Package for the Social Sciences (SPSS: Version 24, Chicago, Illinois, USA) and Microsoft Excel. The measurement error / reliability of the musculoskeletal profiling test battery used in this study was examined using the systematic bias ratio and the random error components of the 95% ratio limits of agreement [29, 30], coefficient of variation based on differences, intraclass correlation (2-way random [inter-rater] or 2-way mixed [intra-rater], single measures, absolute model) [31–33], and t-test or Wilcoxon signed-rank test. The confidence interval (CI) for the ratio limits of agreement was also calculated, in order that the precision of the limits could be evaluated. The intraclass correlation results were presented with descriptive guidelines [< 0.40, poor; 0.40–0.59, fair; 0.60–0.74, good; 0.75–1.00, excellent] [34]. Cohen’s d effects sizes (≤ 0.19, trivial; 0.20–0.49, small; 0.50–0.79, medium; ≥ 0.80, large) were also reported to describe the magnitude of the systematic bias [35]. These statistical methods were selected so that relative (intraclass correlation) and absolute reliability (ratio limits of agreement and coefficient of variation) could be investigated.

Heteroscedasticity was examined (for each of the four test groups) using the correlation of the absolute difference between the two trials of repeated measurements and their mean, on raw and log transformed data. Of the 32 samples analysed in the present study, the correlations for logarithm transformed data was reduced on nine occasions compared to the equivalent raw data samples. An advantage of logarithm transformation is that the measurement error / reliability of the different tests comprising the musculoskeletal profiling test battery can be compared, regardless of the test’s original units of measurement [9, 29, 30]. For completeness and because the values may be more familiar to the reader, the mean and standard deviation calculations for all trials and test orders are presented in the results section, but all reliability analyses were conducted using natural logarithm (base e) data.

The systematic bias ratio and the random error component of the 95% ratio limits of agreement can be used to establish the range of likely variation in a measurement due to error. For those unfamiliar with interpreting the systematic ratio bias and the random error components of the 95% limits of agreement on a ratio scale, if a rater recorded 35.9° for a Supine Medial Hip Rotation test, and the bias and agreement ratios for this test were 1.039 and 1.563 respectively, the calculation would be: 1.039 * 1.563 and 1.039 ÷ 1.563 = 1.624 and 0.665 respectively. Therefore, although the Supine Medial Hip Rotation measure was 35.9°, given the measurement error indicated by the systematic bias and random error components of the limits of agreement, to be 95% certain a subsequent measurement was a true change, it would have to be less than 23.9° (35.9° * 0.665) or greater than 58.3° (35.9° * 1.624). An alternative and slightly less conservative approach (based on 1 standard deviation above the mean as opposed to 2 standard deviations [1.96] above the mean as above) can be taken using a coefficient of variation. For example, if the coefficient of variation was 25.6%, to be 68% certain a subsequent measure was a true change, the corresponding range of variation for the measurement described above would need to be less than 26.7° or greater than 45.1° (35.9° _¯_ /+ [35.9° * 0.256]). Calculations labelled “variationLoA” and “variationCV” respectively have been made and presented in Tables 1 and 2 to highlight to the reader the practical consequences of the measurement error in the musculoskeletal profiling test battery examined in the present study. The alpha level was set at p < 0.05 to determine statistical significance.

#### Categorical measurements

The intra- and inter-rater reliability categorical measurements were evaluated using the weighted kappa, along with the descriptive interpretations for the kappa measurement agreement (< 0.00, poor; 0.00–0.20, slight; 0.21–0.40, fair; 0.41–0.60, moderate; 0.61–0.80, substantial; 0.81–0.99, almost perfect [agreement]), and the percentage agreement between measurements was also evaluated [36, 37].

## Results

### Tests measured in degrees and centimetres

**Fig 4:**
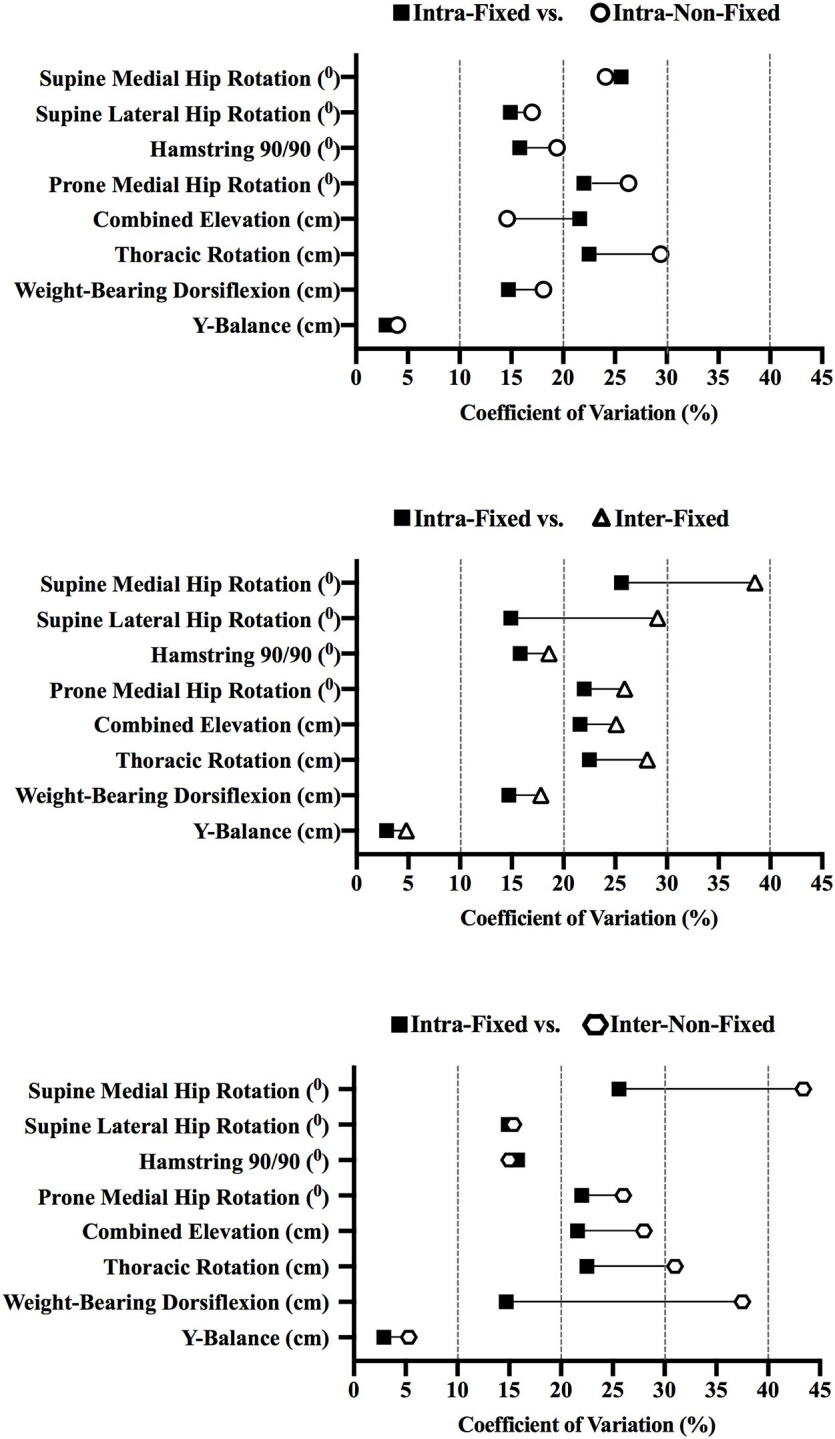
Coefficient of variation for musculoskeletal profiling tests measured in degrees and centimetres.

Tables 1 and 2 show the results for the intra- and inter-rater relative and absolute reliability analyses for the musculoskeletal profiling tests measured in degrees and centimetres. Intraclass correlations (relative reliability) for the intra-rater-fixed order groups ranged from 0.47 (“fair”) to 0.95 (“excellent”), and were the largest for five out of the eight tests measured in degrees and centimetres, while the remaining experimental groups reported smaller intraclass correlation measures: intra-rater-non-fixed order (0.42 [“fair”] to 0.93 [“excellent”]); inter-rater-fixed order (0.04 [“poor”] to 0.88 [“excellent”]); and, inter-rater-non-fixed order (0.20 [“poor”] to 0.82 [“excellent”]). In terms of relative reliability, the intra-rater-fixed order coefficient of variation ranged from 2.9 to 25.6% (see Fig 4), and the ratio limits of agreement from 1.058 (*/÷ 1.020) to 1.563 (*/÷ 1.117) (see Tables 1 and 2), and were smallest for five out of the eight tests measured in degrees and centimetres. The remaining experimental groups reported larger absolute reliability measures for their coefficient of variation and ratio limits of agreement: intra-rater-non-fixed order [4.0 to 29.4%, 1.080 (*/÷ 1.031) to 1.658 (*/÷ 1.219)]; inter-rater-fixed order [4.8 to 38.5%, 1.096 (*/÷ 1.033) to 1.894 (*/÷ 1.245)]; and inter-rater-non-fixed order [5.3 to 43.4%, 1.107 (*/÷ 1.040) to 2.026 (*/÷ 1.319)].

### Categorical measurements

The weighted kappa varied from 0.08 (“slight agreement”) to 0.73 (“substantial agreement”) across the four test order designs, and was 0.51 and 0.47 (“moderate agreement”, Beighton and Lumbar Quadrant respectively) when the tests were conducted in the intra-rater-fixed order (Table 3).

## Discussion

Given the time, effort and resources dedicated to conducting musculoskeletal profiling tests in young elite players in soccer academies, the current study sought to quantify the intra- and inter-rater relative and absolute measurement error / reliability of a battery of musculoskeletal profiling tests used in such a setting, and also sought to examine if the order in which the test battery was administered influenced measurement error / reliability. Relative measurement error / reliability (established using an intraclass correlation) varied from 0.04 (“poor”) to 0.95 (“excellent”), but was largest for five out of the eight tests measured in degrees and centimetres when the intra-rater-fixed test order was adopted (compared to intra-rater-non-fixed, inter-rater-fixed and inter-rater-non-fixed orders). For these eight tests, conducted using the theoretically optimal intra-rater fixed test order, the intraclass correlation was always above 0.70 (“good”), and for all but one test (the Hamstring 90/90) the measurement error / relative reliability would have been categorised as “good” or “excellent”. Generally, the intraclass correlations quantified by the study were higher for the musculoskeletal profiling tests measured in centimetres (0.42–0.95) compared with those measured in degrees (0.04–0.82). In terms of absolute measurement error / reliability (established using a coefficient of variation based on differences, or the ratio limits of agreement), the coefficient of variation ranged from 2.9–43.4%, and the ratio limits of agreement from 1.058–2.026. The coefficient of variation and the ratio limits of agreement were smallest for five out of eight tests measured in degrees and centimetres when the tests were administered in an intra-rater-fixed test order. For the two categorical musculoskeletal profiling tests, the weighted kappa varied from 0.08 (“slight agreement”) to 0.73 (“substantial agreement”) across the four test order designs, and was 0.51 and 0.47 (“moderate agreement”, Beighton and Lumbar Quadrant respectively) when the tests were conducted in the intra-rater-fixed order. Based on the present study, intra- and inter-rater relative and absolute measurement error / reliability varied depending on the particular musculoskeletal profiling test administered, and the order in which the battery of tests was conducted clearly influenced measurement error / reliability.

Previous intra- and inter-rater reliability studies investigating the relative reliability of a musculoskeletal profiling test battery comprising identical or similar tests to those used in the present study, reported intraclass correlations ranging from 0.30 [“poor”] to 0.97 [“excellent”] [4, 11, 12, 22, 23, 25, 38–44]. This wide variation in intra- and inter-rater intraclass correlation values between tests was also evident in the present study (0.04 [“poor”] to 0.95 [“excellent”]). Based on the results for the intra-rater-fixed test order (which generally elicited the highest values) the intraclass correlations for the musculoskeletal tests examined in the current study would have been categorised as “excellent” or “good”, only the Hamstring 90/90 test elicited an intraclass correlation below 0.70, which overall is very positive. When compared to previous reliability studies examining absolute reliability using a coefficient of variation, the present study reported wider coefficient of variation ranges (2.9 to 43.4%) compared to previous findings [3.3 to 12.4%] [4, 22], although the range was smaller when just the intra-rater-fixed test order was considered (2.9 to 25.6%). This smaller range in the previously published research may be a function of a coefficient of variation derived from differences in methodology in the present study, which will tend to produce higher coefficient of variation values. Most previous studies have not utilized the systematic bias ratio and the random error component of the 95% ratio limits of agreement, but generally, for the musculoskeletal tests conducted in the intra-rater-fixed test order, the systematic bias was small (within 2–3%), although the ratio limits ranged 1.06 to 1.56. It has been argued that measurement error / reliability studies should utilize this method, and it has been found that among 13 types of sports medicine and science measurements ratio limits varied from 1.06 to 3.01 [30]. For the categorical measurements (Beighton and Lumbar Quadrant tests) weighted kappa values ranged from 0.08 (“slight agreement”) to 0.73 (“substantial agreement”) in the current study, compared to previous research which reported kappa values ranging from 0.59 (“moderate agreement) to 0.87 (“almost perfect agreement) [28, 45, 46]. The kappa values for the categorical measurements in the current study would appear to report smaller values than previous research studies, but the current study used a “weighted kappa”, which assigns less weight to agreement as categories are further apart [37]. Clearly, there is considerable variation in the reported measurement error / reliability between different musculoskeletal tests in the current study and in the measurement error / reliability reported in previous research. It should be noted that direct study comparison is often not straight-forward as, for example, test order is often not explicitly stated. However, beyond the differences in the populations used in the current study compared to previous research, the variation within and between previous findings and the current study could also potentially be explained by the current study’s combination of having more participants, raters and musculoskeletal tests than previously reported findings. Furthermore, each of the previous studies used their raw data to evaluate relative and absolute reliability, while the present study used natural logarithmically transformed data, which typically produces smaller intraclass correlations and larger coefficients of variations. Therefore, the use of different populations, the inconsistency in reporting testing orders, the varying protocols and data analyses, all potentially explain the variation in reported measurement error among the current and previous research studies examining measurement error / reliability when administering musculoskeletal profiling tests.

It is clear from Tables 1 and 2 and Fig 4 that there are differences in the measurement error values (and therefore potentially in reliability) between the 10 different musculoskeletal tests comprising the battery examined in the current study. For example, the coefficient of variation for the Y-Balance test was the smallest of all the tests measured in degrees or centimetres (ranging from 2.9 to 5.3%), while the same values for the Hamstring 90/90 test ranged from 15.0 to 19.4%, and for the Supine Medial Hip Rotation test ranged from 24.1 to 43.4%. The implications of these differences in measurement error for assessment of reliability in a practical context are discussed below, but clearly, given the range of values discussed in the examples above and presented in detail in Tables 1 and 2 and Fig 4, it would be incorrect for one to suggest that all musculoskeletal test procedures have the same amount of measurement error associated with their administration. In addition, relative measurement error / reliability (established using an intraclass correlation), was largest for five out of the eight tests measured in degrees and centimetres when the intra-rater-fixed test order was adopted (compared to intra-rater-non-fixed, inter-rater-fixed and inter-rater-non-fixed orders). In terms of absolute measurement error / reliability, the coefficient of variation (and the ratio limits of agreement) were smallest for five out of the eight tests measured in degrees and centimetres when the tests were administered in an intra-rater-fixed test order (Supine Lateral Hip Rotation, 14.9; Prone Medial Hip Rotation, 22.0; Thoracic Rotation, 22.5; Weight-Bearing Dorsiflexion, 14.7; Y Balance, 2.9 [%]). What is apparent is that deviating from the intra-rater-fixed test order design generally results in comparatively larger measurement error and hence comparatively poorer reliability. For the two categorical musculoskeletal profiling tests, the weighted kappa was 0.51 and 0.47 (“fair”, Beighton and Lumbar Quadrant respectively) when the tests were conducted in the intra-rater fixed order. Where the intra-rater-fixed test order did not elicit the ‘best’ measurement error values, the difference between the intra-rater-fixed order and the next best value recorded among the three other test designs was very small. In summary, the measurement error varies considerably between the 10 different musculoskeletal profiling tests of which the battery was composed, and this will have implications for how the battery and its component tests may be utilised in practice. The optimal test order in which the musculoskeletal profiling test battery should be conducted is intra-rater-fixed order, and deviating from this design does generally result in comparatively larger measurement error and hence comparatively poorer reliability assessments.

The results of the current study do allow the quantification of the measurement error with respect to the particular musculoskeletal profiling tests examined in the study. An obvious question is: are the tests reliable? Essentially, this is a question of how big is the difference in musculoskeletal function one is trying to measure with a particular test, and what is the magnitude of the measurement error when one is making a measurement with that test. There are a series of scenarios where one might want to use a battery of musculoskeletal profiling tests: to monitor functional change between players within and across age groups; to detect functional characteristics or risk factors that predispose players to injury; to assess changes in function due to injury and rehabilitation; examining asymmetrical differences in musculoskeletal function; and for assessing the effect of training. Answering the question relating to ‘reliability’ means considering whether the musculoskeletal profiling tests examined in the current study are suitable for all or some of these purposes. Reference to some of the data presented in Tables 1 and 2 and Fig 4 should demonstrate how these questions could be addressed. For example, hypothetically, if left and right limb asymmetry was deemed to be evidenced by a 15% difference in the measured values between limbs on completion of all tests in the current battery on a particular player, is the 15% difference evidence of asymmetry in the player? Using the results from the coefficient of variation for the intra-rater-fixed order analysis, the measurement error associated with the Y-Balance and the Weight Bearing Dorsiflexion is likely to be 2.9 and 14.7% respectively, and this means that a difference of 15% between left and right limbs measurements would lie outside these error boundaries, and hence the findings of a 15% difference between the right and left limbs, could be interpreted as indicative of underlying asymmetry with respect to these particular musculoskeletal functions in this particular player. However, for all the other tests in the battery using the intra-rater fixed order strategy (and by implication the musculoskeletal functions that they evaluate), the coefficient of variation was equal to or greater than 15% (ranging from 15% to 25.6%), and so a difference in values between the left and right limbs of 15% in a player could be explained by measurement error and therefore could not be interpreted as indicative of an underlying asymmetry. Similar evaluations could be done for other situations such as changes with age, injury, or training.

In terms of assessing measurement error / reliability, the coefficient of variation is a less conservative approach than the ratio limits of agreement. Basically, the narrower boundaries associated with the coefficient of variation are because the calculations underpinning it are based on one standard deviation, whereas the ratio limits of agreement boundaries arise from calculations using 1.96 or essentially two standard deviations. Using the hypothetical 15% difference between limbs again, and the data from the intra-rater-fixed order design, only when using the Y-Balance test could one be sure that a difference of 15% is not likely to be due to measurement error. (The ratio limits of agreement for the Y-Balance test in the intra-rater fixed order was 1.058, (essentially 5.8%); in the other 9 tests, the ratio limits ranged from 1.309 to 1.563 (essentially 30.9 to 56.3%). This discussion and the results of the current study demonstrate the potential problems of adopting a blanket approach when assessing a characteristic such as asymmetry (or any other characteristic for that matter): one boundary is unlikely to be applicable to all types of situations, given the variation in the measurement error demonstrated in the current study between the 10 different musculoskeletal profiling tests, and given that the error boundaries are clearly dependent on the statistical approach employed to assess measurement error. In addition, it stresses the importance of matching the measurement error associated with a specific test of a particular musculoskeletal function, to the difference one is trying to discern due to factors such as age, injury, training or asymmetry. If the difference is big, even a test with a large measurement error may be adequate for the purpose, and consequently, sufficiently ‘reliable’. Conversely, if the difference one is trying to discern is small, even a test with a small measurement error may be inadequate for the purpose, and consequently, not sufficiently ‘reliable’.

Ideally, testing batteries should be administered using the optimal design, but, in a practical setting, this may not be possible for a variety of pragmatic reasons. The ‘real-world’ challenges of an elite sporting environment such as a soccer academy, involving multiple teams of young performers, often means that it is very difficult to conduct regular or even infrequent ‘testing’ sessions in an optimal way, as time is always limited among players and staff who are committed to very busy training and match schedules, along with non-soccer related obligations that are an integral requirement of involvement in an elite academy. In many academy and soccer environments the number of staff available to help administer testing sessions may also be limited. This study sought to quantify intra- and inter-rater measurement error (and by implication reliability) when one deviates from the theoretically optimum test administration order (“intra-rater-fixed order”) and utilises alternative testing designs (“intra-rater non-fixed”, “inter-rater-fixed” and “inter-rater non-fixed” orders) for a musculoskeletal profiling test battery comprising 10 tests. It is clear from Tables 1 and 2 and Fig 4 that the theoretically optimal testing design (“intra-rater-fixed order”) does generally elicit the smallest measurement error. But, Tables 1 and 2 and Fig 4 also make clear that deviating from this ideal testing design does result in greater measurement error, which would mean that when investigating musculoskeletal function and age, injury, training or asymmetry in young, elite academy soccer players, detecting genuine change would be more difficult if the optimal testing design (“intra-rater-fixed order”) were not adopted.

It should be acknowledged that a limitation of the current study was that the number of measurements recorded within each experimental condition were uneven. Originally, 104 participants volunteered for the study but, due to injury, illness, international soccer duty and other unexpected factors, only 75 players took part and were tested on two occasions. Additionally, there was a medical emergency on one of the testing occasions, so the raters had to be rearranged for some tests. In addition, there would have been training sessions between testing occasions as it was not possible to prevent these elite players from training as the study was conducted during the competitive season, and consequently these training sessions could have influenced the study’s findings. Another potential limitation of the current study was that no account was taken of chronological age or biological maturity (although inter-individual variation in musculoskeletal function is as likely to be present within groups as it is to be present between groups) and future research should perhaps seek to expand upon the current study by aligning musculoskeletal profiling measurements to chronological age and biological maturity.

## Conclusions

In summary, given the time, effort and resources dedicated to conducting musculoskeletal profiling tests in young, elite players in soccer academies, the present study sought to quantify the relative and absolute measurement error / reliability of a battery of musculoskeletal profiling tests used in such a setting, and also sought to examine if the order in which the test battery was administered, and who it was administered by, influenced measurement error / reliability. In terms of relative and absolute measurement error / reliability, there was considerable variation in the intraclass correlation, coefficient of variation and ratio limits of agreement when the individual tests were compared, even within the intra-rater-fixed test order, although the intra-rater-fixed test order generally elicited the smallest measurement errors when compared to the other test order designs. Clearly, the type of musculoskeletal profiling test administered and the order in which a battery of such tests are conducted influences measurement error and hence their reliability. This variation, and the statistical methods used to examine measurement error / reliability, needs to be carefully considered when musculoskeletal profiling tests are being used for practical purposes such as investigating musculoskeletal function and age, injury, training or asymmetry in young, elite academy soccer players.

## Supporting information

S1 Table Distribution of participants based on age group within the four test groups. (DOCX)

S2 Table Participant characteristics of each of the four test groups (mean ± standard deviation). (DOCX)
