# Supplementary material for: Reliability of a musculoskeletal profiling test battery in elite academy soccer players
Source: PLoS One. 2020 Jul 23;15(7):e0236341. doi: 10.1371/journal.pone.0236341 (PMC7377398; doi:10.1371/journal.pone.0236341)
Supplement: S1 Table — (DOCX) [file pone.0236341.s001.docx]

**S1 Table.** Distribution of participants based on age group within the four test groups.

| **Age Group** | **Group A**  **(n)** | **Group B**  **(n)** | **Group C**  **(n)** | **Group D**  **(n)** |
| --- | --- | --- | --- | --- |
| **12** | 4 | 4 | 2 | 2 |
| **13** | 5 | 4 | 3 | 2 |
| **14** | 4 | 3 | 2 | 2 |
| **16** | 5 | 5 | 3 | 2 |
| **18** | 4 | 4 | 1 | 1 |
| **23** | 4 | 4 | 3 | 2 |
| **Total** | 26 | 24 | 14 | 11 |
