# Supplementary material for: Reliability of a musculoskeletal profiling test battery in elite academy soccer players
Source: PLoS One. 2020 Jul 23;15(7):e0236341. doi: 10.1371/journal.pone.0236341 (PMC7377398; doi:10.1371/journal.pone.0236341)
Supplement: S2 Table — (DOCX) [file pone.0236341.s002.docx]

**S2 Table.** Participant characteristics of each of the four test groups (mean ± standard deviation).

| **Age Group** | **Group A** | **Group B** | **Group C** | **Group D** |
| --- | --- | --- | --- | --- |
| Age (decimal years) | 15.4 ± 2.3 | 15.2 ± 2.0 | 15.2 ± 2.1 | 15.0 ± 2.3 |
| Stature  (m) | 1.685 ± 0.120 | 1.687 ± 0.119 | 1.705 ± 0.122 | 1.722 ± 0.125 |
| Body mass (kg) | 59.0 ± 13.6 | 59.4 ± 13.1 | 60.7 ± 16.5 | 63.4 ± 13.6 |
